# Disease gene prioritization with quantum walks

**DOI:** 10.1093/bioinformatics/btae513

**Published:** 2024-08-22

**Authors:** Harto Saarinen, Mark Goldsmith, Rui-Sheng Wang, Joseph Loscalzo, Sabrina Maniscalco

**Affiliations:** Algorithmiq Ltd, FI-00160 Helsinki, Finland; Department of Mathematics and Statistics, Complex Systems Research Group, University of Turku, FI-20014, Turku, Finland; Algorithmiq Ltd, FI-00160 Helsinki, Finland; Department of Mathematics and Statistics, Complex Systems Research Group, University of Turku, FI-20014, Turku, Finland; Department of Medicine, Brigham and Women’s Hospital, Boston, MA 02115, United States; Department of Medicine, Brigham and Women’s Hospital, Boston, MA 02115, United States; Algorithmiq Ltd, FI-00160 Helsinki, Finland

## Abstract

**Motivation:**

Disease gene prioritization methods assign scores to genes or proteins according to their likely relevance for a given disease based on a provided set of seed genes. This scoring can be used to find new biologically relevant genes or proteins for many diseases. Although methods based on classical random walks have proven to yield competitive results, quantum walk methods have not been explored to this end.

**Results:**

We propose a new algorithm for disease gene prioritization based on continuous-time quantum walks using the adjacency matrix of a protein–protein interaction (PPI) network. We demonstrate the success of our proposed quantum walk method by comparing it to several well-known gene prioritization methods on three disease sets, across seven different PPI networks. In order to compare these methods, we use cross-validation and examine the mean reciprocal ranks of recall and average precision values. We further validate our method by performing an enrichment analysis of the predicted genes for coronary artery disease.

**Availability and implementation:**

The data and code for the methods can be accessed at https://github.com/markgolds/qdgp.

## 1 Introduction

The utilization of network modelling has proven to be an effective technique for studying the structure and dynamics of biological systems (Stelzl and Wanker 2006, Yan *et al.* 2017). Consequently, there has been an increasing effort through biophysical and high-throughput methods to form protein–protein interaction (PPI) networks that consist of the physical and/or functional interactions between human proteins. This type of complex network, called the human interactome, sets the basis for the field of network medicine (Barabási *et al.* 2011, Loscalzo and Barabasi 2011, Lee and Loscalzo 2019).

One of the main propositions of network medicine is that a disease phenotype is rarely a consequence of abnormal effects in a product of a single gene, but, rather, the effects are scattered across multiple gene products interacting in the human interactome (Kwang-Il *et al.* 2007, Menche *et al.* 2015). These interacting proteins associated with a given disease thus form a subnetwork and are then expected to gather in a local neighbourhood in the human interactome (Barabási *et al.* 2011). As the proteins in these disease subnetworks are collectively involved in the development or progression of a disease, they offer key insights into the underlying molecular mechanisms and biological processes driving the disease. However, understanding these molecular disease mechanisms can be time-consuming and require significant resources when using high-quality and/or small-scale experiments. Thus, locating these disease neighbourhoods, called disease modules (Loscalzo and Barabasi 2011), has been a major challenge in the field with a clear need for improved computational methods.

In the context of disease module identification, the typical scenario involves a predefined set of proteins known as seed proteins, which have been carefully curated and experimentally validated for their association with a specific disease. However, these seed proteins often form an incomplete subnetwork that does not fully represent the expected disease module. The primary objective is to predict the entire disease module by leveraging the structure of the PPI network and the seed proteins. This problem is commonly referred to as disease gene prioritization, as the aim is to systematically incorporate additional proteins into the module based on their likelihood of being disease-associated. Once the seed genes are identified and mapped to the PPI, network-based connector proteins provide the missing links needed to define the disease module. Thus, by using computational methods and network analysis techniques, researchers can enhance the comprehensiveness of the disease module and identify potential candidate genes for further experimental investigation.

Because the network approach to diseases has already demonstrated its effectiveness for multiple diseases (Petrochilos *et al.* 2013, Sharma *et al.* 2015, Lee *et al.* 2020, Wang *et al.* 2021, Kumar and Ramanathan 2023, Pandey and Loscalzo 2023), it is of utmost importance to develop and elaborate on methods that identify disease modules. Unfortunately, there are a few well-known challenges in identifying the disease modules. On the one hand, the PPI networks tend to be very incomplete, with various estimates suggesting that they account for approximately 20–30% of the total connections within the interactome (Hart *et al.* 2006, Venkatesan *et al.* 2009, Marc *et al.* 2011, Luck *et al.* 2020). Hence, the structure of the human interactome is partly unknown and the disease modules tend to be more scattered around the interactome than is expected. Furthermore, it was recently shown in Lazareva *et al.* (2021) that many disease module prediction methods do not perform better on PPI networks than on random networks with the same node degrees (see Supplementary Section 0.6 for a preliminary analysis using our method). On the other hand, as noted in Ghiassian *et al.* (2015), the state of the art community detection algorithms, which are shown to work well in other network clustering tasks, tend to perform very poorly in locating these disease modules. Hence, there has been increasing effort applied to the development of methods that are specifically designed to infer the disease modules in these very incomplete networks.

We introduce a novel method centred around quantum walks on the interactome. Continuous-time quantum walks, initially proposed in Farhi and Gutmann (1998), are the quantum analogues of continuous-time classical random walks, which describe the propagation of a particle over a graph. Together with their discrete-time counterpart (Aharonov *et al.* 1993), they have received much attention for their applications in quantum information processing (Kempe 2003, Venegas-Andraca 2012), quantum computation (Childs 2009), and quantum transport (Mülken and Blumen 2011). While the methods that we describe here are *quantum-inspired*, since they are implemented classically, we can foresee that these algorithms will be even more efficient if run on quantum devices. Continuous-time quantum walks have already been implemented on various physical platforms (Manouchehri and Wang 2014), including optical setups (Peruzzo *et al.* 2010, Preiss *et al.* 2015, Tang *et al.* 2018, Wang *et al.* 2020, Young *et al.* 2022) and superconducting devices (Yan *et al.* 2019, Gong *et al.* 2021), and they can also be simulated on gate-based quantum computers (Qiang *et al.* 2016, Loke and Wang 2017). Nevertheless, there are still challenges in implementing quantum walks on near-term quantum devices due to the large number of qubits required for running quantum walks on PPI networks. Given the current limitations of qubit decoherence and noise, the implementation of large-scale continuous-time quantum walks on a quantum device still requires several more years of hardware and algorithmic improvements. However, the quantum walks described here can be run efficiently on classical computers (see Supplementary Section 0.6 for a discussion of running times).

In general, random walk methods are known to perform well in a variety of tasks (Xia *et al.* 2020), and have also been used for disease module detection (Köhler *et al.* 2008, Li and Patra 2010, Xie *et al.* 2012, Petrochilos *et al.* 2013, Joodaki *et al.* 2021, Gentili *et al.* 2022). However, most of these methods are based on discrete-time random walks on the network or its modifications, while their continuous-time counterparts have not been studied as extensively, even though they seem to have rather competitive performance (Köhler *et al.* 2008). Quantum walks have not been previously used in disease module identification. Based on these observations, we propose a new disease gene prioritization method based on continuous-time quantum walks using the PPI adjacency matrix (QA). The choice of continuous-time quantum walks is 2-fold. Firstly, quantum walks have been shown to perform competitively in other network applications such as link prediction (Qian *et al.* 2017, Goldsmith *et al.* 2023, Moutinho *et al.* 2023) and spatial search (Malmi *et al.* 2022). Secondly, quantum walks can work analogously to continuous-time classical random walk methods (such as the diffusion kernel) (Köhler *et al.* 2008), described in Supplementary Section S0.1, but offer more flexibility in terms of the dynamics that they can produce, which allows them to be modified suitably for the disease gene prioritization task.

In disease module identification and prioritization problems, evaluating the performance of different methods is not straightforward since the ground truth of the predictions is unknown. In this study, we use a standard cross-validation procedure as is typically found in the machine learning and the link prediction literature (see, e.g. Hastie *et al.* 2009, Lü and Zhou 2011, and references therein), to compare the performance of our proposed method against a disease module detection algorithm (DIAMOnD) (Ghiassian *et al.* 2015), neighbourhood scoring (NBR) (Navlakha and Kingsford 2010), random walk with restart (RWR) (Köhler *et al.* 2008), and diffusion kernel (DK) (Köhler *et al.* 2008) methods. These methods are described in Supplementary Section S0.1. We evaluate the methods on seven PPI networks from various sources and three different datasets from different databases for disease seeds. However, to compare the methods in this manner, they must be able to yield an arbitrary number of gene scores. Many algorithms such as SCA (Wang and Loscalzo 2018), TOPAS (Buzzao *et al.* 2022), and DOMINO (Levi *et al.* 2021) use Steiner trees or other ways of connecting the seed proteins so that the size of the predicted module varies greatly from disease to disease. Importantly, it is not a hyperparameter that the user can control. Thus, our cross-validation-based comparison is not feasible for these methods. In addition, all of those methods aim to form a single necessarily connected module for all diseases, which might ignore crucial disease components (Agrawal *et al.* 2018).

Our study makes several significant contributions to the existing literature. Firstly, we propose a novel method based on quantum walks and showcase its superior performance compared to state-of-the-art algorithms. Secondly, we establish the robustness of our method by evaluating its performance across seven PPI networks and three distinct disease gene datasets, utilizing multiple evaluation metrics within a cross-validation framework. Furthermore, we validate the biological relevance of the predicted genes by conducting a case study on coronary artery disease.

## 2 Materials and methods

### 2.1 The setup

Consider a PPI network modelled by an undirected graph G=(V,E), where *V* is the set of proteins (nodes) of size *n* and *E* is the set of interactions (edges). The *adjacency matrix* of *G* is the *n *×* n* matrix defined by

A=(Aij)={1, if (i,j)∈E,0, if (i,j)∈E.

The *network Laplacian* is defined as L=D−A, where *D* is the diagonal *degree matrix* given by $D=\text{diag}\left(\sum_{j}A_{1j},\ldots ,\sum_{j}A_{nj}\right)$.

A *disease module DM* in the network *G* is a (connected) subnetwork of *G* that contains proteins $S=(s_{1},\ldots ,s_{d})$ called *seed proteins*. The seed proteins should be understood as a set of proteins that by definition are part of the disease module while the rest of the module DM∖S is unknown. Thus, the problem of locating the disease module *DM* of unknown size is to find the proteins in *G* associated with a disease given a set of seed proteins *S*.

### 2.2 Continuous-time quantum walks

In the classical continuous-time random walk on a network every edge of the network is associated with an independent Poisson process with unit intensity. When the walker is at some node, it will remain there until one of the Poisson processes at a neighbouring edge jumps, at which point the walker follows that edge to the corresponding neighbour, and then the process repeats. Working out the mathematical details leads to a rather simple closed-form formula for the evolution of the walker.

In contrast to a classical random walk, a quantum walk on a network evolves according to the laws of quantum physics and its evolution is governed by the Schrödinger equation. Consequently, the paths of the walker across the network can interfere constructively or destructively. This interference can cause the evolution of the quantum walker to be significantly different from the classical one (Aharonov *et al.* 1993, Childs *et al.* 2002).

A continuous-time quantum walk (Farhi and Gutmann 1998) on a graph *G* is defined by considering the Hilbert space H spanned by the orthonormal vectors $\{|i\rangle {\}}_{i=1}^{n}$, corresponding to the nodes of the network, and the unitary transformation $e^{-\mathrm{itH}},$ where *H* is the Hamiltonian that is based on the structure of the network under consideration. Using this unitary transformation, the initial state vector |ψ(0)〉 in H evolves via

(1) $$|\psi (t)\rangle =e^{-\mathrm{itH}}|\psi (0)\rangle .$$

In general, the Hamiltonian *H* can be any Hermitian matrix related to *G* as long as it describes the structure of the network (Venegas-Andraca 2012), but usually the Laplacian *L* or the network adjacency matrix *A* is used (Wong *et al.* 2016). This is in contrast to the classical case, where the Laplacian must be used, giving the quantum walk more flexibility in terms of the dynamics. In this paper, we exploit this property by modifying the adjacency matrix by adding a constant real number *α* to the diagonals corresponding to the given seed proteins. We note that this is equivalent to adding *α* self-edges at the seed proteins in the network and, consequently, it increases the likelihood of the walker remaining in the vicinity of the seed nodes for a longer period of time (see the ablation study in the Supplementary section for details). This effect is very similar to lazy classical random walks. Thus, as the Hamiltonian we use

(2) $$A_{S}=A+\alpha \;\text{diag}(v_{S}),$$

where *v_S_* is a binary vector defined by $v_{i}=1_{\left\{i\in S\right\}}$, where *S* is the set of seed proteins and 1 is the indicator function. In the Supplementary Section C, we explore why setting *α* to a small, positive value may offer an increase in performance.

In order to obtain a probability transition matrix from the Hamiltonian, we evolve the system for a time *t* and perform a measurement, which can be done by taking the square of the modulus of the entries of the unitary operator $e^{-itA_{S}},$ where $i=\sqrt{-1}.$ The entries of the probability transition matrix are

(3) $$P_{uv}(t)=|\langle v|e^{-itA_{S}}|u\rangle {|}^{2}.$$

Note that, contrary to the classical case, where randomness comes from stochastic transitions between states, state transitions are deterministic in the quantum walk, with randomness resulting from the measurement and collapse of the wave function.

Once these transition probabilities are calculated, we proceed similarly to the diffusion kernel method initially proposed in Köhler *et al.* (2008), which postulates that a protein is more likely to be associated with a disease if the walker is likely to transition from that protein to any of the seed proteins. Thus the likelihood score $L_{t}(v)$ for protein *v* is computed by summing the probabilities for the walker to move from *v* to any node in the seed set *S*, computed at time *t*. More specifically,

$$L_{t}(v)=\sum_{s\in S}P_{vs}(t).$$

In this case, *t* is a hyperparameter that can be chosen for the dataset in question.

When considering a specific disease in the disease module identification task, we do not need the whole matrix exponential, but, rather, its action on the seed vector *v_S_*. Consequently, calculating the scores for all the considered networks can be an efficient process (Higham and Al-Mohy 2010, Al-Mohy and Higham 2011).

### 2.3 Data

#### 2.3.1 Human interactome networks

We tested our methods on variety of different human PPI networks, which have previously been used for disease module detection. The *GMB* PPI was constructed from seven different sources, described in Menche *et al.* (2015); the *WL* PPI integrated data from PPIs, protein complexes, kinase–substrate interactions, and signalling pathways (Wang and Loscalzo 2021); and the 5 PPI networks *BioGRID*, *STRING*, *APID*, *HPRD*, and *IID* were retrieved from well-known PPI databases and made available in Lazareva *et al.* (2021).

Some statistics of these networks are listed below in Table 1. We observe that the networks have high clustering and that they are very sparse. Furthermore, the networks are approximately scale-free (Barabási and Albert 1999), which is typical of biological networks. One distinguishing feature of PPI networks compared to most other complex networks is that they may sometimes contain self-edges, which represent the ability of a protein to interact with itself.

**Table 1.** Properties of the networks that were tested.

Network	|V|	|E|	〈k〉	ρ	*C*	*A*	SIPs
HPRD	8498	33 935	7.987	0.001	0.109	−0.034	0
GMB	13 329	141 150	21.179	0.002	0.174	0.115	2794
APID	14 257	292 964	41.098	0.003	0.122	−0.046	7
BioGRID	15 400	237 045	30.785	0.002	0.104	−0.063	2
STRING	15 821	387 175	48.944	0.003	0.407	0.182	7
IID	16 280	314 956	38.692	0.002	0.116	−0.065	4063
WL	17 491	354 640	40.551	0.002	0.082	−0.034	0

|V|: number of nodes; |E|: number of edges; 〈k〉: average degree; ρ: network density; C: average clustering coefficient; A: assortativity; SIPs: number of self-interacting proteins.

#### 2.3.2 Disease genes

We gathered disease data from three main sources: Open Targets (OT) (Ochoa *et al.* 2022), DisGeNET (DGN) (Piñero *et al.* 2020), and the disease data provided in Ghiassian *et al.* (2015) (GMB). Open Targets and DisGeNET are large-scale databases that integrate data from a combination of different sources such as GWAS databases, genetics, drugs, animal models, and the scientific literature. The GMB dataset from Ghiassian *et al.* (2015) was curated by experts from OMIM (Hamosh *et al.* 2002, Mottaz *et al.* 2008) and the PheGenI database (Ramos *et al.* 2014).

The Open Targets and DisGeNET sources each include thousands of diseases, while the GMB dataset from Ghiassian *et al.* (2015) only contains 70 expert selected diseases. In order to use more reliable disease sets from Open Targets and DisGeNET, we filtered the disease sets using a ranking (score) of the disease gene associations provided by these datasets. For Open Targets, we only used disease-gene associations with a score of at least 0.6; for DisGeNET we only used disease-gene associations with a score of at least 0.3 (so that there is likely at least one ‘curated’ source), and ensured that seed genes have a disease specificity index of at least 0.5. Finally, for each PPI, we only used the diseases whose PPI coverage contains at least 15 genes after the above filtering so that sufficient structure remains in the seed network after seed removal during the cross-validation process. Table 2 shows the number of diseases for each dataset, on each PPI network considered.

**Table 2.** Number of diseases for disease sets and networks.

Network Disease set	APID	BioGRID	GMB	HPRD	IID	STRING	WL
DGN	358	354	333	263	380	379	379
GMB	64	63	65	58	64	63	64
OT	49	49	48	31	50	50	49

## 3 Results

In order to assess the performance of our method, we selected four other disease gene prioritization methods previously considered in the literature for comparison: Diffusion kernel (DK) (Köhler *et al.* 2008), random walk with restart (RWR) (Köhler *et al.* 2008), DIAMOnD (Dia) (Ghiassian *et al.* 2015), and neighbourhood scoring (NEI) (Navlakha and Kingsford 2010). These methods, along with the used hyperparameter choices, are described in more detail in the Supplementary Section A. For QA the value *t *=* *0.45 is chosen using grid search on the GMB network and disease set, and the value *α* = 5 is chosen based on the analysis done in Supplementary Section C. In principle, these values can be chosen for each network and disease separately, but we leave these fixed across all the diseases and networks to demonstrate the robustness of the method.

**Figure 1.**
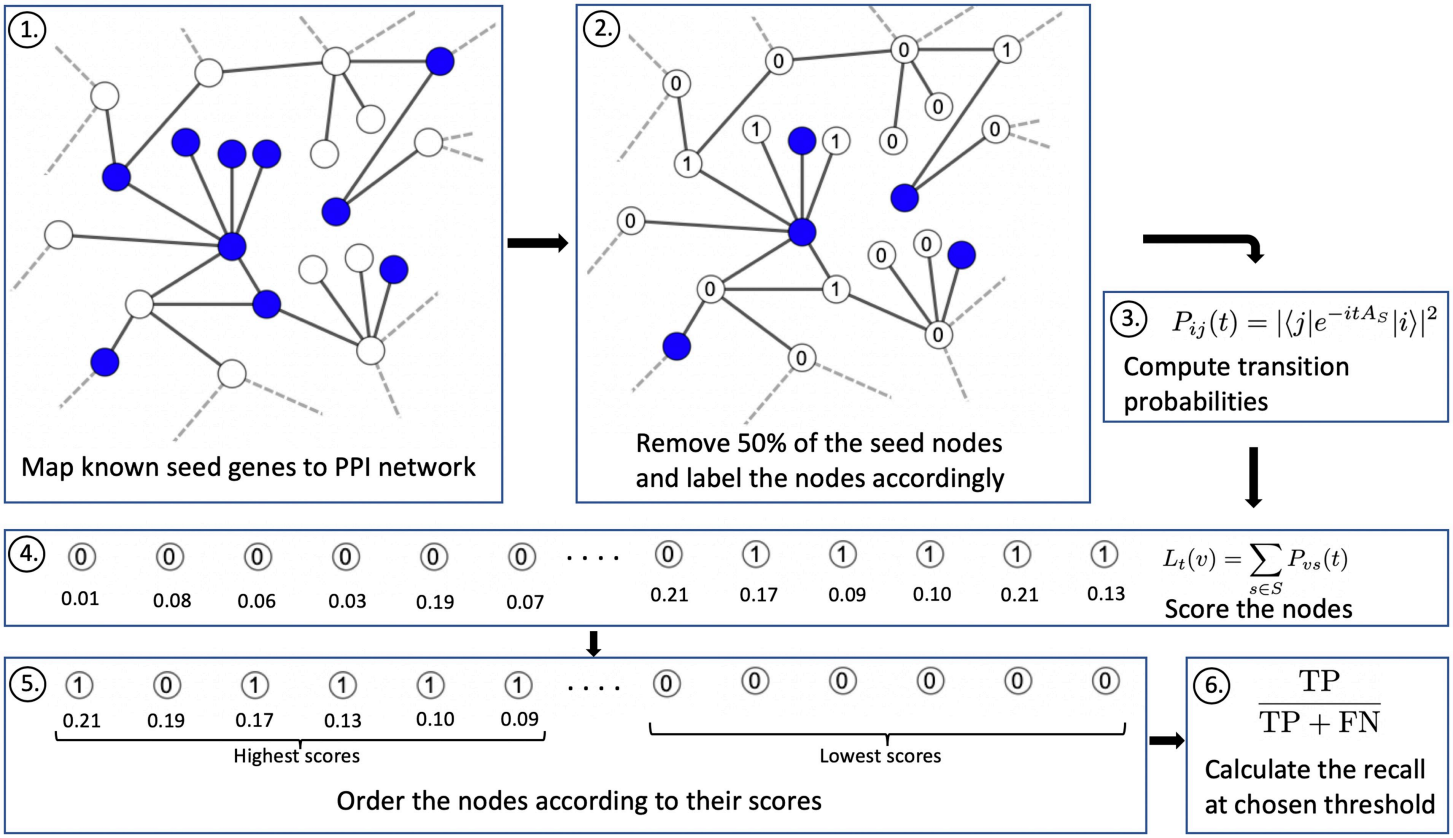
Description of the algorithm and evaluation procedure: (1) The seed genes are mapped to a given PPI network. (2) Half of the seed nodes are randomly selected and labelled as nonseeds. (3) Transition probabilities of the quantum walk are calculated for every pair of nodes in the network. (4) Genes are scored according to their seed node transition probabilities. (5) The genes are ranked from highest to lowest scores, with their ground truth labels preserved. (6) The recall value for a given threshold is calculated as the fraction of true seeds in the top *N* scores (we use *N *=* *25 and *N *=* *300 in our results).

Since the ground truth of the disease modules is unknown, we tested the algorithms using cross-validation. For each disease, we randomly removed 50% of the seed genes, and then sorted the nonseeds in descending order according to their scores given by each method. Recall values were then calculated based on this ranking. However, there is considerable variance in the recall values across different diseases, making the averaging of these values a less robust metric for measuring the performance of the methods across diseases. Therefore, to ensure a more comprehensive comparison of the methods across diseases, we calculated the mean reciprocal ranks (MRR) based on the recall values, similar to Agrawal *et al.* (2018). For completeness, we also calculated average precisions and their corresponding mean reciprocal ranks for a subset of the models in Supplementary Tables S3 and S4. However, since average precision requires a ranking of all nodes in the network rather than just the top *N*, we chose to focus on mean reciprocal ranks in terms of recall for two reasons: (i) in practical applications the number of genes that can be considered for a more detailed anaylsis is limited, so we are typically only interested in knowing the top *N* genes; (ii) it is not computationally feasible to rank all of the nodes in the network using the DIAMOnD algorithm. The cross-validation process was repeated 10 times for each disease, and the results were averaged. The whole pipeline can be seen below in Fig. 1. A more detailed description of the cross-validation procedure and the metrics used is provided in the Supplementary Section B. Furthermore, we found that our results were similar for other seed gene removal fractions (see Supplementary Table S5).

Tables 3 and 4 present the average MRR values achieved over ten runs for each method across all considered disease sets and networks, specifically ranking the top 25 and top 300 nodes, respectively. Note that column averages were calculated before rounding the MRR values to three decimal places.

**Table 3.** Average values of mean reciprocal ranks for each model on each disease set and network, after 25 nodes are scored. The bolded values highlight the best performing method in each row.^a^

	QA	DIA	DK	NBR	RWR
DGN					
APID	0.695 (0.31)	**0.700** (0.35)	0.666 (0.33)	0.488 (0.32)	0.677 (0.28)
BioGRID	**0.721** (0.31)	0.714 (0.32)	0.648 (0.33)	0.533 (0.32)	0.664 (0.30)
GMB	**0.756** (0.31)	0.650 (0.34)	0.696 (0.32)	0.500 (0.33)	0.641 (0.30)
HPRD	**0.771** (0.30)	0.633 (0.36)	0.744 (0.31)	0.578 (0.36)	0.716 (0.31)
IID	**0.705** (0.31)	0.693 (0.34)	0.655 (0.33)	0.489 (0.32)	0.666 (0.29)
STRING	0.618 (0.33)	**0.660** (0.34)	0.426 (0.27)	0.394 (0.25)	0.574 (0.26)
WL	**0.693** (0.31)	0.673 (0.34)	0.666 (0.33)	0.524 (0.34)	0.671 (0.29)
GMB					
APID	**0.743** (0.30)	0.644 (0.36)	0.560 (0.32)	0.399 (0.28)	0.715 (0.27)
BioGRID	**0.682** (0.30)	0.653 (0.35)	0.545 (0.31)	0.468 (0.32)	0.599 (0.26)
GMB	**0.719** (0.32)	0.506 (0.32)	0.623 (0.32)	0.356 (0.26)	0.640 (0.30)
HPRD	**0.709** (0.33)	0.555 (0.33)	0.667 (0.34)	0.450 (0.32)	0.635 (0.29)
IID	**0.751** (0.30)	0.567 (0.36)	0.558 (0.31)	0.359 (0.24)	0.614 (0.27)
STRING	**0.662** (0.32)	0.573 (0.32)	0.333 (0.20)	0.332 (0.20)	0.585 (0.28)
WL	0.612 (0.32)	0.604 (0.36)	0.492 (0.30)	0.341 (0.22)	**0.637** (0.28)
OT					
APID	**0.640** (0.33)	0.534 (0.33)	0.427 (0.26)	0.268 (0.09)	0.530 (0.25)
BioGRID	0.582 (0.29)	**0.612** (0.35)	0.541 (0.35)	0.328 (0.22)	0.571 (0.28)
GMB	**0.706** (0.33)	0.538 (0.31)	0.519 (0.28)	0.339 (0.24)	0.489 (0.25)
HPRD	**0.774** (0.27)	0.491 (0.30)	0.583 (0.33)	0.332 (0.24)	0.446 (0.24)
IID	**0.677** (0.33)	0.557 (0.34)	0.393 (0.23)	0.271 (0.09)	0.524 (0.25)
STRING	0.581 (0.34)	**0.646** (0.33)	0.322 (0.20)	0.257 (0.06)	0.527 (0.24)
WL	**0.623** (0.31)	0.529 (0.33)	0.468 (0.33)	0.329 (0.22)	0.526 (0.26)
Average	**0.687**	0.606	0.549	0.397	0.602

a Standard deviations are shown in parentheses.

**Table 4.** Average values of mean reciprocal ranks for each model on each disease set and network, after 300 nodes are scored. The bolded values highlight the best performing method in each row.^a^

	QA	DIA	DK	NBR	RWR
DGN					
APID	**0.623** (0.30)	0.535 (0.34)	0.501 (0.32)	0.340 (0.21)	0.462 (0.25)
BioGRID	**0.606** (0.30)	0.533 (0.33)	0.506 (0.33)	0.371 (0.24)	0.510 (0.26)
GMB	**0.638** (0.32)	0.426 (0.28)	0.592 (0.32)	0.373 (0.26)	0.504 (0.26)
HPRD	0.595 (0.31)	0.415 (0.29)	**0.640** (0.30)	0.322 (0.23)	0.526 (0.29)
IID	**0.643** (0.31)	0.510 (0.32)	0.509 (0.33)	0.356 (0.23)	0.506 (0.28)
STRING	**0.712** (0.32)	0.390 (0.27)	0.389 (0.28)	0.397 (0.24)	0.558 (0.27)
WL	**0.573** (0.30)	0.491 (0.32)	0.547 (0.34)	0.360 (0.22)	0.475 (0.25)
GMB					
APID	**0.626** (0.32)	0.510 (0.33)	0.432 (0.29)	0.338 (0.23)	0.499 (0.27)
BioGRID	**0.561** (0.30)	0.526 (0.32)	0.475 (0.32)	0.331 (0.21)	0.527 (0.30)
GMB	**0.631** (0.33)	0.381 (0.26)	0.500 (0.33)	0.378 (0.26)	0.591 (0.28)
HPRD	0.531 (0.28)	0.368 (0.25)	**0.624** (0.34)	0.299 (0.22)	0.614 (0.30)
IID	**0.617** (0.30)	0.435 (0.27)	0.481 (0.33)	0.329 (0.22)	0.522 (0.27)
STRING	**0.788** (0.29)	0.289 (0.10)	0.297 (0.21)	0.366 (0.18)	0.634 (0.27)
WL	**0.672** (0.32)	0.468 (0.30)	0.397 (0.27)	0.323 (0.17)	0.555 (0.30)
OT					
APID	**0.611** (0.29)	0.491 (0.32)	0.474 (0.32)	0.290 (0.22)	0.510 (0.29)
BioGRID	0.515 (0.23)	0.503 (0.33)	**0.546** (0.36)	0.288 (0.18)	0.509 (0.26)
GMB	**0.622** (0.31)	0.420 (0.29)	0.547 (0.30)	0.312 (0.24)	0.514 (0.26)
HPRD	**0.672** (0.31)	0.317 (0.15)	0.542 (0.32)	0.218 (0.04)	0.597 (0.27)
IID	**0.643** (0.29)	0.418 (0.29)	0.539 (0.35)	0.277 (0.17)	0.470 (0.25)
STRING	**0.777** (0.28)	0.353 (0.26)	0.350 (0.26)	0.346 (0.23)	0.653 (0.28)
WL	**0.626** (0.29)	0.430 (0.30)	0.514 (0.34)	0.307 (0.19)	0.417 (0.20)
Average	**0.632**	0.439	0.495	0.330	0.531

a Standard deviations are shown in parentheses.

The QA model consistently outperforms other models across most disease sets and networks at both 25 and 300 ranked nodes, as indicated by the highest MRR values in most cases. The DIA model also demonstrates competitive performance in a few cases when 25 proteins are ranked. The performance of the other models is mixed, and they do not consistently outperform other models for any disease set and network combination.

In the Supplementary Section D, we show a more global view of these results, plotting MRR as a function of the number of ranked nodes ranging from 1 to 300. We plot the average MRR values obtained from the ten runs for all diseases in the three disease sets across the seven networks. The conclusion remains the same: The QA model very consistently outperforms other models when about 100 or more nodes are ranked and remains on par with other models when using <100 nodes.

We also include sample running times in Supplementary Table S6 of the Supplementary Section E. We merely note here that QA has comparable running times to the already existing methods we compared it against and that all the methods are relatively fast when evaluating a single disease on a single network.

In summary, the QA model outperforms other models in terms of average mean reciprocal rank. DIA, RWR, and DK also demonstrate good performance in some cases, but do not consistently outperform the QA model, especially when more than 100 nodes are ranked. Overall, the QA model appears to be the most robust and effective model for the given tasks. A similar conclusion is arrived at when considering the recall values (see the Supplementary Tables S1 and S2). Similar conclusions can be drawn when ranking the models based on average precision instead of recall, as shown in Supplementary Table S4.

### 3.1 Coronary artery disease

To further validate our methods, we used coronary artery disease (CAD) as a case study to demonstrate that the disease genes prioritized by the QA model are biologically relevant. We compiled a set of 81 seed genes for CAD derived from a meta-analysis of large-scale genome-wide association studies (GWAS) (Nikpay *et al.* 2015), mapped them to the WL human interactome (the largest of PPIs considered here), and used the QA model to prioritize disease genes for CAD. Of the 81 seed genes, 73 were be found in the WL human interactome. For this seed set, we optimized the parameters for the QA model using grid search and found that *t *=* *0.11 and *α* = 5 yield the best recall in cross-validation. Using these parameters we then considered the top 200 prioritized genes by QA. We also prioritized genes from the same starting seed set with the other models in this paper and found that of the top 200 genes prioritized by QA, 79 are not prioritized by any of the other methods.

**Figure 2.**
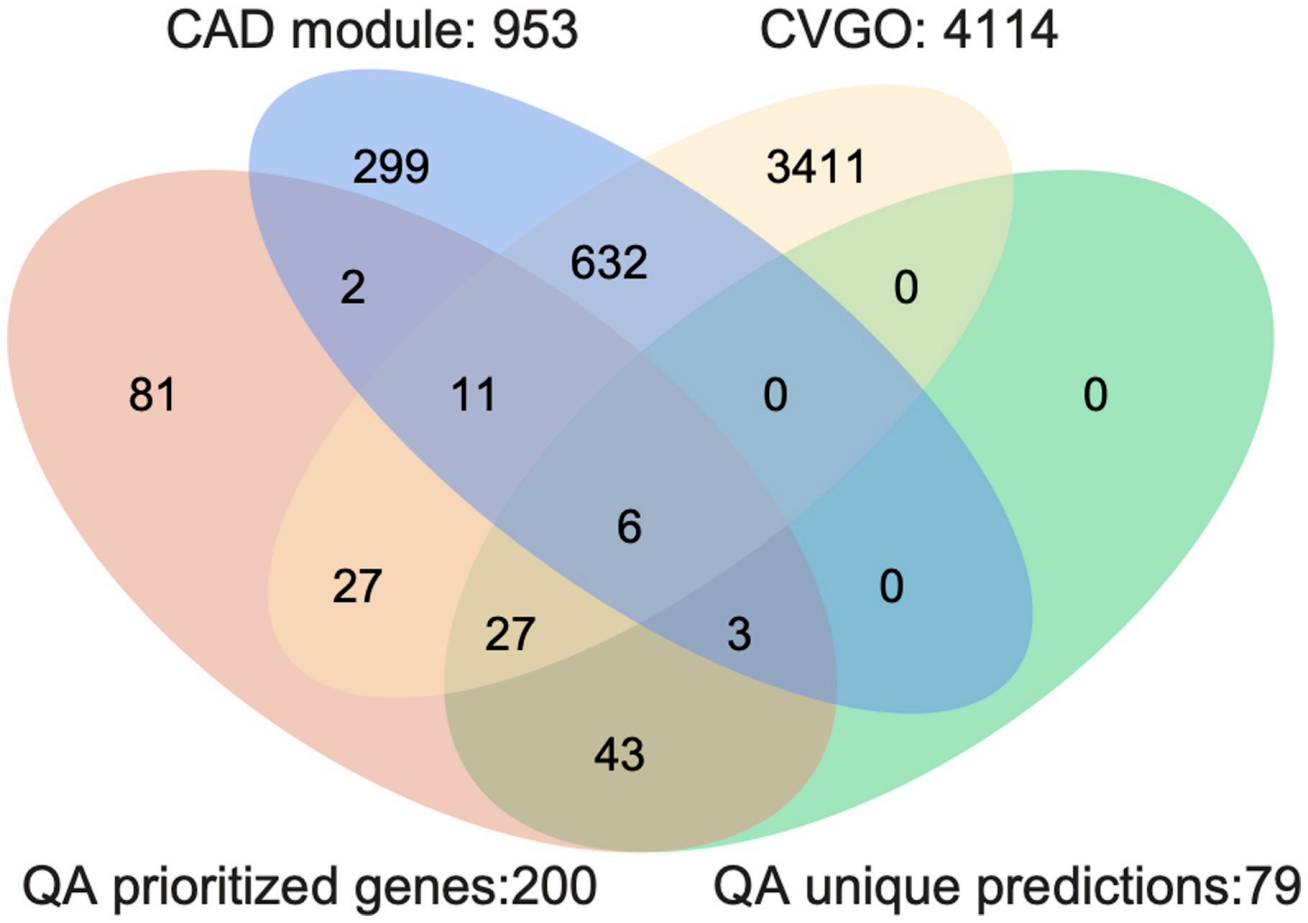
Overlap of the top 200 genes and 79 prioritized genes that were unique to QA with the CAD module compiled from OpenTargets, DisGeNet, and the genes from Cardiovascular Gene Ontology (CVGO) Annotation Initiative.

We then examined the overlap of the top 200 genes and 79 prioritized genes that were unique to QA with the CAD module compiled from OpenTargets, DisGeNet, and the genes from Cardiovascular Gene Ontology (CVGO) Annotation Initiative (https://www.ebi.ac.uk/GOA/CVI). The top 200 prioritized genes and 79 uniquely predicted genes by the QA module have 22 and 9 overlapping genes with the CAD module, respectively (hypergeometric test, *P *<* *0.001 and *P *<* *0.027). These predictions are significantly enriched with genes from CVGO (hypergeometric test, *P *<* *8.1×10^−5^ and *P *<* *2.4×10^−4^ respectively for the top 200 predictions and 79 unique predictions) (Fig. 2). Among these prioritized genes are included ApoC2 [PMID: 26044596], Nox1 [PMID: 21411092], and IL1beta [PMID: 33362770], demonstrating that based on only a small number of seed genes from GWAS studies, QA was able to prioritize genes that have biological relevance to cardiovascular (patho)biology.

## 4 Conclusion

Our study introduces a novel algorithm for disease gene prioritization based on continuous-time quantum walks on PPI networks. The proposed algorithm demonstrates great performance compared to several well-known gene prioritization methods across multiple disease sets and various PPI networks. By encoding self-loops for the seed nodes into the underlying Hamiltonian, the quantum walker was shown to remain more local to the seed nodes, leading to improved performance.

The results indicate that the quantum walk-based algorithm can effectively prioritize disease genes by leveraging the structure of the PPI network and the known seed genes. The continuous-time quantum walk approach provides a flexible and efficient alternative to classical random walk methods more commonly used in various network biology tasks. However, further research and validation are necessary to fully understand the potential of quantum walks and their applicability to other biological network-related tasks. For example, performing simulation studies to design causal connections would increase our understanding of quantum walks on these networks. This question is however left for future research.

Overall, the study contributes to the growing field of network medicine and computational methods for disease gene prioritization, highlighting the value of incorporating quantum-inspired algorithms in biological network analysis. With advances in quantum computing, future applications of quantum walks in this domain may hold even greater promise.

## Supplementary Material

btae513_Supplementary_Data

## Data Availability

The code, PPI networks, and disease datasets needed to reproduce the cross-validation results are available at https://github.com/markgolds/qdgp.
