# Supplementary material for: Disease gene prioritization with quantum walks
Source: Bioinformatics. 2024 Aug 22;40(8):btae513. doi: 10.1093/bioinformatics/btae513 (PMC11361815; doi:10.1093/bioinformatics/btae513)
Supplement: btae513_Supplementary_Data [file btae513_supplementary_data.pdf]

Bioinformatics, 2022, pp. 1–3

doi: DOI HERE
Advance Access Publication Date: Day Month Year
Paper

PAPER

Supplementary: Disease Gene Prioritization With Quantum Walks

Harto Saarinen,[1,*] Mark Goldsmith,[2,*] Rui-Sheng Wang,[3] Joseph Loscalzo[4] and Sabrina Maniscalco[5]

[1, 2, 5]Algorithmiq Ltd, Kanavakatu 3, FI-00160, Helsinki, Finland, [1, 2]Complex Systems Research Group, University of Turku, Department of Mathematics and Statistics, FI - 20014, Turku, Finland and [3]Department of Medicine, Brigham and Women's Hospital, Harvard Medical, Street, MA 02115, Boston, United States of America
[*]Corresponding author. hoasaa@utu.fi and margol@utu.fi
FOR PUBLISHER ONLY Received on Date Month Year; revised on Date Month Year; accepted on Date Month Year

Abstract

Supplementary material for the original article disease gene prioritization with quantum walks.

Related works

Diffusion kernel (DK). Köhler et al. (2008) A continuous-time classical random walk on a network is a Markov process with state space V characterized by a rate matrix L and initial distribution $\mathbf{p}(0)$ over a set of nodes. Hence, the dynamics are governed by the *diffusion kernel*

$$\mathbf{p}(t) = \mathbf{p}(0)e^{-tL}. \qquad (1)$$

These transition probabilities are then used to compute scores for proteins that are not in the seed set by calculating

$$S(i) = \sum_{s \in S} P_{is}.$$

This model has a single hyperparameter, t. For our experiments, we used $t = 0.3$ since this value provided the strongest results on the GMB network and data set. No discussion of the setting of this hyperparameter was provided in Köhler et al. (2008). We also note following Köhler et al. (2008) that using these small values for t the random walker behaves similar to a lazy random walk.

Random walk with restart (RWR). Köhler et al. (2008) The random walk with restart is a discrete time random walk, where at every step there is a probability of returning to the initial state. The initial state is chosen to be a uniform distribution at the seed proteins, and the scores for proteins are their probability values at the steady-state distribution. In this model, the restart probability can be considered as a hyperparameter, however, we found that our results were not sensitive to it. We used a restart probability of 0.4 for all experiments, as was done in Ghiassian et al. (2015).

DIAMOnD (DIA). Ghiassian et al. (2015) DIAMOnD iteratively adds proteins to the disease module based on their connectivity significance to the seed proteins. Because DIAMOnD expands the module one protein at the time, it can rank proteins away from the immediate neighbourhood of the disease proteins. For our experiments, we used the extended version of the algorithm described in Ghiassian et al. (2015), weighting the seed proteins with a value of $\alpha = 9$ (setting $\alpha \approx 10$ was recommended in the original paper).

Neighbourhood scoring (NEI). Navlakha and Kingsford (2010) In the neighbourhood method, each protein is assigned a score that is proportional to the percentage of its neighbours associated with the disease. Thus, this method is limited to scoring only the immediate neighbourhood of the seed proteins as all other proteins are given a score of zero.

Metrics

Since the ground truth of the disease modules is unknown, we tested the algorithms using cross-validation. For each disease, we randomly removed 50% of the seed genes, and reserved these genes as positive test cases. The rest of the genes were used as negative testing data. In other words, after removing the 50% of the seed genes, the non-seeds were ranked by sorting them in descending order according to their scores given by each method, and the genes with higher scores were deemed most likely to exist. This ranking was then compared to the evaluation set to see how well the positive test cases were ranked. This process was repeated 10 times for each disease, and the results were averaged (see below).

In order to compare the protein rankings of the methods under consideration, we used recall defined as

$$\text{True positive rate} = \text{Recall} = \frac{\text{TP}}{\text{TP} + \text{FN}},$$

where TP = true positive and FN = false negative. To calculate recall from the rankings, a threshold that serves as

© The Author 2022. Published by Oxford University Press. All rights reserved. For permissions, please e-mail: journals.permissions@oup.com

a cut-off rule has to be selected (the predictions above the thresholds are classified as positive and below it as negative). We consider thresholds up to 300, which means that the top 300 predictions are evaluated (In the Figures this number is referred as iterations).

There are, however, very large differences in the recall values across different diseases (see the standard deviations in the Tables 1 and 2), because some of the disease seed sets are more easily predicted by all methods, while other are much more difficult. This leads to a large variance in the recall distributions, and thus makes averaging of the recall values over the diseases a less robust metric for measuring the performance of the methods. Therefore, to ensure a more comprehensive comparison of the methods across diseases, we calculated the mean reciprocal ranks, as was done in Agrawal et al. (2018). This approach provides a more reliable way to evaluate the method's performance in a diverse range of scenarios.

For a method k evaluated on a particular set of diseases $\mathcal{D}$, the mean reciprocal rank is defined by

$$\mathrm{MMR}(k) = \frac{1}{|\mathcal{D}|} \sum_{d=1}^{|\mathcal{D}|} \frac{1}{R_d^k},$$

where $|\mathcal{D}|$ is the number of diseases in the set and R_d^k is the rank of the kth method for disease d, relative to the average recalls (over 10 trials) of the other methods being considered. In this way, the number of diseases for which the highest recalls are achieved can be compared, rather than the raw recall values.

It is also worth noting that we do not use the area under the receiving-operator characteristics curve or average precision as our main metrics for comparing different algorithms as is customary in binary classification tasks. The reasons are threefold. Firstly, these metrics have high variance due to the wide spectrum of diseases under consideration, as was discussed above. Secondly, they consider the scores of the entire node set, but in practice we are typically only interested in the highest ranking nodes. Thirdly, it is too costly for the DIAMOnD algorithm to assign scores to every protein in the network, making it impossible to compare it against the other methods. Nevertheless, we have included average precisions for all the other methods in Table 3, defined as

$$AP = \sum_n (\mathrm{Recall}(n) - \mathrm{Recall}(n-1))\mathrm{Precision}(n),$$

where n sums over all the scored nodes in the network, $\mathrm{Precision}(n) = \mathrm{TP}/(\mathrm{TP}+\mathrm{FP})$ is the precision, and $\mathrm{Recall}(n)$ is the recall when threshold is set to n.

Ablation study on seed diagonals

The modified Hamiltonian, A_S, defined by

$$A_S = A + \alpha \, \mathrm{diag}(v_S),$$

describes our use of the hyperparameter α, which has the effect of adding α self-edges to seed nodes before the quantum walks are performed. In this section, we explore the effect this hyperparameter has on the walk dynamics.

First, we demonstrate that our choice of $\alpha = 5$ does, indeed, enhance the performance of our method by comparing the case used in our results ($\alpha = 5$) against the version of our model where seed diagonals are not treated in any particular way, i.e. $\alpha = 0$. We compare these variations on the GMB disease set and network. The mean reciprocal ranks are shown in Figure 1, and their average recalls are compared in Figure 2.

While Figures 1 and 2 show that setting values to the seed diagonals does, indeed, improve overall performance, they do not offer any explanation as to why this should be the case. To this end, we offer the following hypothesis: a positive value of α allows the walker to remain more local for low-degree nodes. To justify this claim, we examine the mean distance travelled from seed node s after time t, defined as

$$\mu_s(t) = \sum_v d_{sv} P_{sv}(t), \qquad (2)$$

where d_{sv} is the shortest path length from node s to node v, and $P_{sv}(t)$ is the transition probability from s to v after time t, defined by

$$P_{uv}(t) = |\langle v| e^{-itA_S} |u\rangle|^2.$$

In Figure 3, we show the results of the following experiment, conducted on the GMB PPI network: we choose a random low/medium/high degree node s (defined as nodes with degrees in the range $[1, 10]$, $[50, 60]$, $[200, 300]$, respectively) to use as a single starting seed node, then we compute the mean distance travelled from s for multiple values of t in the range $[0, 1]$, and for four different settings of the hyperparameter α. This process is repeated 50 times for each of the 3 degree ranges, and the results are averaged over the 50 runs, resulting in 4 curves for each of the α settings.

Two main conclusions can be drawn from Figure 3. Firstly, settings $\alpha = 0$ and $\alpha = 5$ have similar mean distance travelled curves for medium and high degree nodes, but not for low degree nodes, agreeing with our above hypothesis. Secondly, the highest value of α considered reduces the mean distance travelled in all cases. Thus, the setting of this hyperparameter should be chosen carefully, otherwise the walker may not have a chance to explore regions of the network not immediately adjacent to any seeds.

The previous experiment considered the quantum walk dynamics for a single starting node. Next, we examined the more relevant case of having multiple seed nodes. More specifically, we choose four arbitrary diseases from the GMB disease set, and we compute the mean distance travelled for each disease by averaging the resulting mean distance travelled curves over the seeds for each disease. In other words, for a disease with seed set S, we compute

$$\frac{1}{|S|} \sum_{s \in S} \mu_s(t)$$

for several values of t. The results are shown in Figure 4. Indeed, we can see that, on average, quantum walkers will travel farther for $\alpha = 0$ when compared to our setting of $\alpha = 5$.

Recall results and additional plots

Table 1 and Table 2 show the average recalls of the methods considered when ranking the top 25 and top 300 scores, respectively. A more detailed view of the mean reciprocal ranks and recalls are shown as a function of the top 300 rankings in Figure 5 and Figure 6, respectively.

Running times

We compare the running times for the algorithms considered here in Table 6. For all methods except DIA, we show running times for calculating scores for all nodes in the WL PPI network

for coronary artery disease as found in the GMB data set. For DIA, we compute only the top 500 scores.

The computational complexity for QA depends on various factors. The bottleneck for our method is calculating the matrix exponential, which can be done using approximate Taylor polynomials Bader et al. (2019). Thus, the problem reduces to a constant number of matrix multiplications that can typically be solved more quickly than the naive $O(n^3)$ methods (where n is the number of nodes in the network), for example using Strassen's algorithm or its variations Strassen (1969). However, it is worth noting that when when computing predictions for a single disease using the QA method, it is not necessary to compute the full probability transition matrix resulting from the quantum walk; we need only compute the action of the (sparse) Hamiltonian on a seed indicator vector for each seed in the seed set (see `https://github.com/markgolds/qdgp` for implementation details and Al-Mohy and Higham (2011) for the algorithm that computes the action of a matrix exponential). In contrast, in situations where the PPI network is fixed and predictions for many diseases are required, it may be more efficient to calculate the full probability transition matrix one single time and use it to score the genes in the network for each disease using this pre-computed matrix.

Sensitivity to network randomization

It was recently shown in Lazareva et al. (2021) that many disease module prediction methods do not perform better on PPI networks than on random networks with the same node degrees. In order to test this claim on our method, we adapted code from `https://github.com/dbblumenthal/amim-test-suite` and compared the QA method on two networks (GMB from Ghiassian et al. (2015) and the HPRD network made available in Lazareva et al. (2021)) and 3 corresponding randomized networks (rewired, expected degree, and uniform). In Figure 7, we show QA and DIA's predictive performance for Huntington's disease on GMB and HPRD, as well as their randomized instances. For each model, we computed the top 100 predicted genes. As shown in the Figure, QA does indeed perform significantly better on the original PPI networks than on the randomized ones. We also checked the impact of network randomization on functional relevance by comparing predicted genes with DisGeNET, however there was insufficient overlap to draw to any conclusions of the effect of randomization in this case. The network randomization methods, phenotype information, and mutual information metric details can be found Lazareva et al. (2021).

References

M. Agrawal, M. Zitnik, and J. Leskovec. *Large-scale analysis of disease pathways in the human interactome*, chapter 23, pages 111–122. World Scientific Publishing Co. Pte. Ltd., 2018.

A. H. Al-Mohy and N. J. Higham. Computing the action of the matrix exponential, with an application to exponential integrators. *SIAM journal on scientific computing*, 33(2): 488–511, 2011.

P. Bader, S. Blanes, and F. Casas. Computing the matrix exponential with an optimized taylor polynomial approximation. *Mathematics*, 7(12):1174, 2019.

S. D. Ghiassian, J. Menche, and A.-L. Barabási. A disease module detection (diamond) algorithm derived from a systematic analysis of connectivity patterns of disease proteins in the human interactome. *PLOS Computational Biology*, 11(4):1–21, 04 2015.

S. Köhler, S. Bauer, D. Horn, and P. N. Robinson. Walking the interactome for prioritization of candidate disease genes. *The American Journal of Human Genetics*, 82(4):949–958, 2008. ISSN 0002-9297.

O. Lazareva, J. Baumbach, M. List, and D. B. Blumenthal. On the limits of active module identification. *Briefings in Bioinformatics*, 22(5):bbab066, 2021.

S. Navlakha and C. Kingsford. The power of protein interaction networks for associating genes with diseases. *Bioinformatics (Oxford, England)*, 26:1057–63, 2010.

V. Strassen. Gaussian elimination is not optimal. *Numerische Mathematik*, 13(4):354–356, 1969.

		QA	DIA	DK	NBR	RWR
DGN	APID	0.046 (0.11)	**0.057** (0.13)	0.041 (0.11)	0.030 (0.09)	0.056 (0.12)
	BioGRID	0.038 (0.09)	**0.046** (0.12)	0.031 (0.09)	0.027 (0.08)	0.044 (0.11)
	GMB	0.045 (0.10)	0.050 (0.12)	0.049 (0.11)	0.035 (0.09)	**0.051** (0.11)
	HPRD	0.044 (0.09)	0.034 (0.08)	**0.044** (0.10)	0.030 (0.07)	0.041 (0.10)
	IID	0.046 (0.10)	**0.055** (0.13)	0.042 (0.10)	0.028 (0.09)	0.054 (0.12)
	STRING	0.094 (0.14)	**0.113** (0.18)	0.063 (0.13)	0.075 (0.15)	0.106 (0.18)
	WL	0.038 (0.08)	**0.044** (0.10)	0.033 (0.07)	0.024 (0.06)	0.043 (0.09)
GMB	APID	0.074 (0.12)	0.079 (0.14)	0.057 (0.10)	0.038 (0.08)	**0.080** (0.13)
	BioGRID	**0.066** (0.12)	0.061 (0.11)	0.049 (0.09)	0.032 (0.07)	0.061 (0.11)
	GMB	**0.107** (0.14)	0.089 (0.13)	0.098 (0.14)	0.064 (0.12)	0.103 (0.14)
	HPRD	**0.111** (0.14)	0.101 (0.14)	0.110 (0.15)	0.083 (0.13)	0.110 (0.15)
	IID	**0.080** (0.12)	0.070 (0.13)	0.058 (0.09)	0.038 (0.07)	0.076 (0.13)
	STRING	0.136 (0.14)	**0.157** (0.17)	0.078 (0.11)	0.087 (0.13)	0.148 (0.18)
	WL	**0.072** (0.11)	0.063 (0.11)	0.051 (0.07)	0.036 (0.07)	0.071 (0.11)
OT	APID	0.135 (0.13)	**0.144** (0.16)	0.102 (0.13)	0.085 (0.13)	0.143 (0.16)
	BioGRID	0.106 (0.11)	**0.119** (0.15)	0.086 (0.11)	0.061 (0.10)	0.111 (0.14)
	GMB	**0.145** (0.13)	0.142 (0.14)	0.132 (0.14)	0.098 (0.14)	0.137 (0.15)
	HPRD	**0.137** (0.12)	0.113 (0.11)	0.103 (0.11)	0.061 (0.08)	0.100 (0.11)
	IID	**0.124** (0.11)	0.124 (0.15)	0.081 (0.11)	0.063 (0.11)	0.121 (0.15)
	STRING	0.220 (0.17)	**0.265** (0.22)	0.134 (0.19)	0.138 (0.18)	0.261 (0.22)
	WL	**0.139** (0.13)	0.129 (0.15)	0.097 (0.12)	0.072 (0.11)	0.123 (0.14)
	Average	0.095	**0.098**	0.073	0.057	0.097

Table 1. Average recall values when 25 nodes are scored. Standard deviations are shown in parentheses.

		QA	DIA	DK	NBR	RWR
DGN	APID	**0.164** (0.19)	0.147 (0.19)	0.135 (0.18)	0.118 (0.17)	0.148 (0.19)
	BioGRID	**0.133** (0.17)	0.120 (0.17)	0.105 (0.16)	0.101 (0.16)	0.125 (0.18)
	GMB	**0.163** (0.17)	0.125 (0.15)	0.158 (0.18)	0.114 (0.15)	0.157 (0.18)
	HPRD	0.155 (0.14)	0.121 (0.11)	**0.160** (0.15)	0.095 (0.10)	0.154 (0.15)
	IID	**0.156** (0.18)	0.134 (0.18)	0.127 (0.17)	0.113 (0.17)	0.142 (0.19)
	STRING	**0.299** (0.25)	0.235 (0.23)	0.224 (0.24)	0.252 (0.24)	0.278 (0.25)
	WL	**0.137** (0.17)	0.116 (0.17)	0.119 (0.15)	0.101 (0.16)	0.125 (0.17)
GMB	APID	**0.226** (0.21)	0.209 (0.20)	0.178 (0.20)	0.173 (0.20)	0.214 (0.21)
	BioGRID	**0.194** (0.19)	0.187 (0.18)	0.160 (0.19)	0.154 (0.19)	0.186 (0.20)
	GMB	0.259 (0.23)	0.219 (0.21)	0.234 (0.24)	0.215 (0.21)	**0.261** (0.23)
	HPRD	0.268 (0.20)	0.223 (0.18)	0.260 (0.20)	0.195 (0.16)	**0.273** (0.20)
	IID	**0.227** (0.20)	0.193 (0.19)	0.182 (0.20)	0.171 (0.19)	0.212 (0.20)
	STRING	**0.426** (0.27)	0.309 (0.24)	0.273 (0.25)	0.346 (0.26)	0.408 (0.27)
	WL	**0.194** (0.17)	0.164 (0.16)	0.154 (0.17)	0.156 (0.17)	0.185 (0.18)
OT	APID	**0.400** (0.22)	0.355 (0.24)	0.303 (0.24)	0.236 (0.22)	0.360 (0.23)
	BioGRID	**0.319** (0.22)	0.282 (0.24)	0.265 (0.23)	0.180 (0.20)	0.314 (0.22)
	GMB	**0.416** (0.24)	0.342 (0.23)	0.353 (0.23)	0.241 (0.21)	0.388 (0.23)
	HPRD	**0.445** (0.20)	0.349 (0.21)	0.387 (0.19)	0.216 (0.17)	0.424 (0.20)
	IID	**0.373** (0.22)	0.319 (0.23)	0.291 (0.23)	0.216 (0.21)	0.333 (0.22)
	STRING	**0.655** (0.23)	0.532 (0.28)	0.458 (0.28)	0.501 (0.27)	0.625 (0.24)
	WL	**0.374** (0.23)	0.311 (0.24)	0.297 (0.23)	0.230 (0.20)	0.336 (0.22)
	Average	**0.285**	0.238	0.230	0.196	0.269

Table 2. Average recall values when 300 nodes are scored. Standard deviations are shown in parentheses.

		QA	DK	NBR	RWR
DGN	APID	0.025 (0.07)	0.021 (0.07)	0.015 (0.05)	**0.029** (0.08)
	BioGRID	0.020 (0.06)	0.017 (0.06)	0.012 (0.04)	**0.025** (0.07)
	GMB	0.026 (0.07)	0.026 (0.07)	0.014 (0.04)	**0.027** (0.07)
	HPRD	**0.023** (0.06)	0.022 (0.06)	0.011 (0.03)	0.021 (0.05)
	IID	0.024 (0.07)	0.019 (0.06)	0.012 (0.04)	**0.027** (0.08)
	STRING	0.048 (0.08)	0.031 (0.06)	0.030 (0.06)	**0.053** (0.10)
	WL	0.018 (0.04)	0.014 (0.03)	0.010 (0.03)	**0.022** (0.05)
GMB	APID	**0.044** (0.08)	0.028 (0.05)	0.016 (0.04)	0.041 (0.09)
	BioGRID	**0.035** (0.07)	0.024 (0.05)	0.013 (0.02)	0.031 (0.07)
	GMB	**0.069** (0.11)	0.060 (0.11)	0.027 (0.06)	0.064 (0.11)
	HPRD	**0.064** (0.10)	0.060 (0.10)	0.029 (0.05)	0.061 (0.10)
	IID	**0.045** (0.08)	0.031 (0.05)	0.016 (0.03)	0.043 (0.08)
	STRING	0.075 (0.08)	0.039 (0.05)	0.038 (0.05)	**0.086** (0.11)
	WL	0.030 (0.06)	0.019 (0.04)	0.014 (0.03)	**0.031** (0.06)
OT	APID	0.065 (0.07)	0.042 (0.06)	0.035 (0.07)	**0.075** (0.12)
	BioGRID	0.052 (0.07)	0.042 (0.07)	0.027 (0.05)	**0.065** (0.11)
	GMB	**0.084** (0.09)	0.069 (0.10)	0.041 (0.08)	0.079 (0.11)
	HPRD	**0.071** (0.07)	0.048 (0.06)	0.020 (0.03)	0.051 (0.06)
	IID	0.058 (0.07)	0.038 (0.05)	0.028 (0.06)	**0.066** (0.11)
	STRING	0.123 (0.11)	0.074 (0.11)	0.075 (0.11)	**0.155** (0.18)
	WL	0.058 (0.06)	0.044 (0.06)	0.033 (0.06)	**0.068** (0.11)
	Average	0.050	0.037	0.025	**0.053**

Table 3. Averages values of average precisions, with standard deviations shown in parentheses. Note that the diamond model was left out from this analysis since it is infeasible to run the algorithm to score all nodes in the PPI network.

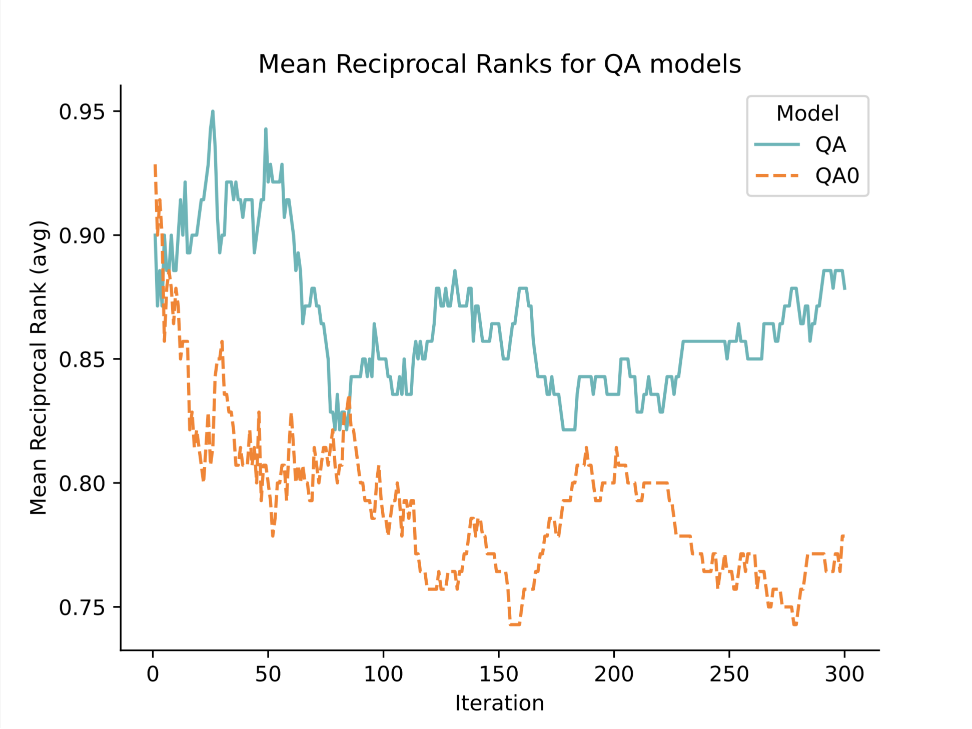

Fig. 1. Mean reciprocal ranks averaged over 10 runs and all diseases in the GMB data set, using the GMB network. Here QA refers to the quantum walk algorithm with 5 placed at seed diagonals (as considered in the main text), and QA0 simply uses the network adjacency matrix as the Hamiltonian.

		QA	DK	NBR	RWR
DGN	APID	**0.618** (0.32)	0.457 (0.28)	0.255 (0.13)	0.500 (0.24)
	BioGRID	**0.612** (0.30)	0.500 (0.31)	0.252 (0.11)	0.468 (0.22)
	GMB	**0.642** (0.31)	0.568 (0.30)	0.237 (0.10)	0.469 (0.22)
	HPRD	0.604 (0.31)	**0.663** (0.29)	0.222 (0.07)	0.441 (0.20)
	IID	**0.632** (0.31)	0.491 (0.30)	0.237 (0.08)	0.483 (0.22)
	STRING	**0.640** (0.32)	0.343 (0.21)	0.254 (0.10)	0.512 (0.22)
	WL	**0.618** (0.32)	0.502 (0.30)	0.269 (0.16)	0.465 (0.19)
GMB	APID	**0.690** (0.32)	0.349 (0.20)	0.226 (0.05)	0.503 (0.20)
	BioGRID	**0.633** (0.32)	0.394 (0.24)	0.231 (0.06)	0.525 (0.23)
	GMB	**0.669** (0.33)	0.449 (0.23)	0.225 (0.04)	0.514 (0.24)
	HPRD	**0.661** (0.31)	0.526 (0.30)	0.230 (0.12)	0.482 (0.23)
	IID	**0.670** (0.30)	0.417 (0.27)	0.227 (0.03)	0.483 (0.21)
	STRING	**0.638** (0.32)	0.300 (0.18)	0.235 (0.03)	0.553 (0.24)
	WL	**0.616** (0.32)	0.367 (0.25)	0.250 (0.12)	0.499 (0.20)
OT	APID	**0.690** (0.33)	0.349 (0.18)	0.231 (0.06)	0.456 (0.20)
	BioGRID	**0.543** (0.26)	0.502 (0.33)	0.235 (0.06)	0.464 (0.22)
	GMB	**0.726** (0.33)	0.431 (0.23)	0.219 (0.04)	0.460 (0.24)
	HPRD	**0.755** (0.28)	0.500 (0.29)	0.203 (0.01)	0.384 (0.14)
	IID	**0.667** (0.31)	0.377 (0.24)	0.232 (0.05)	0.468 (0.23)
	STRING	**0.568** (0.32)	0.268 (0.13)	0.245 (0.05)	0.520 (0.21)
	WL	0.548 (0.30)	0.382 (0.25)	0.254 (0.07)	**0.564** (0.29)
	Average	**0.640**	0.435	0.237	0.486

Table 4. Mean reciprocal ranks based on average precisions, with standard deviations shown in parentheses. As for Table 3, the diamond model was left out from this analysis since it is infeasible to run the algorithm to score all nodes in the PPI network.

	QA	DIA	DK	NBR	RWR
MRR@25	**0.750** (0.30)	0.641 (0.36)	0.688 (0.31)	0.473 (0.34)	0.741 (0.30)
MRR@300	0.673 (0.34)	0.510 (0.31)	0.546 (0.34)	0.452 (0.29)	**0.685** (0.29)
Recall@25	**0.126** (0.22)	0.103 (0.20)	0.113 (0.21)	0.062 (0.16)	0.123 (0.21)
Recall@300	0.297 (0.31)	0.264 (0.30)	0.277 (0.31)	0.257 (0.30)	**0.310** (0.31)
Avg. prec.	**0.051** (0.11)	—	0.044 (0.10)	0.014 (0.03)	**0.051** (0.12)

Table 5. Cross-validation statistics on the GMB PPI network and disease data set when using 90% of the seeds for doing cross-validation (as opposed to 50% in the main text). Statistics are taken over 10 runs.

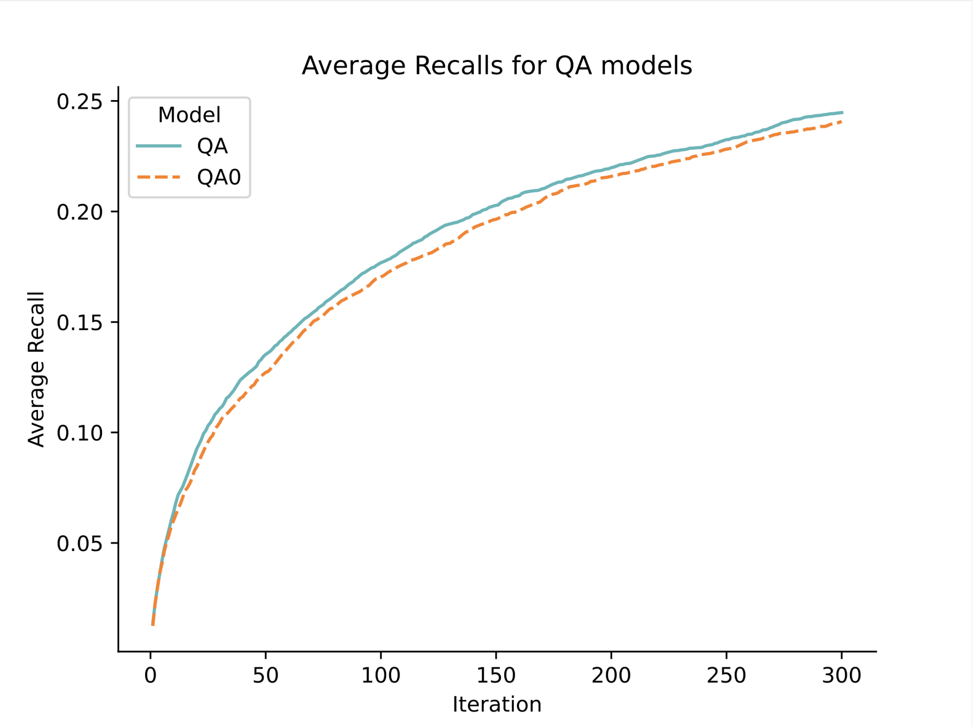

Fig. 2. Recalls averaged over 10 runs and all diseases in the GMB data set, using the GMB network. As in the previous figure, QA refers to the quantum walk algorithm with 5 placed at seed diagonals (as considered in the main text), and QA0 simply uses the network adjacency matrix as the Hamiltonian.

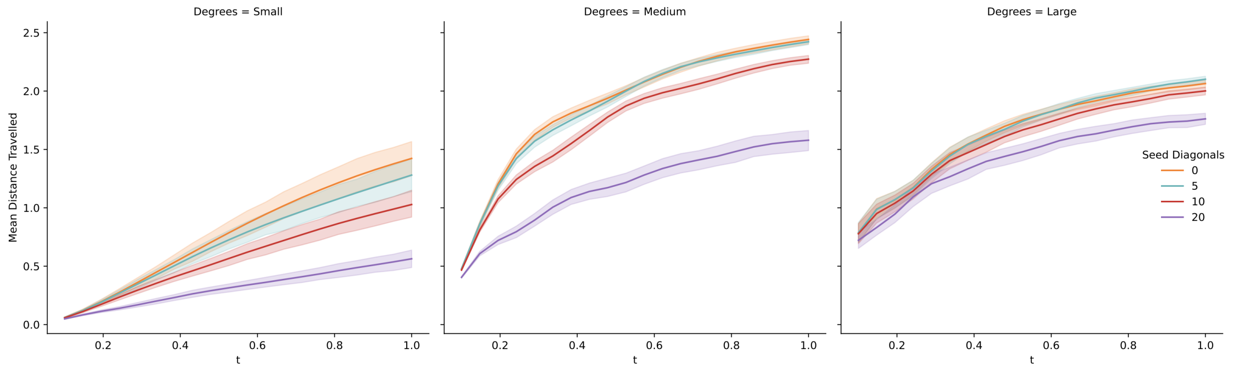

Fig. 3. Mean distance travelled for various settings of the seed diagonals in the QA model for 3 different starting seeds: low (starting seed has degree at most 10); medium (starting seed has degree in $[50, 60]$); large (starting seed has degree in $[200, 300]$). In each of the 3 degree settings, curves are averaged over 50 starting seed runs.

Model	Time (s)
QA	10.768 (0.19)
DIA	19.892 (0.14)
DK	1.078 (0.05)
NEI	1.077 (0.06)
RWR	0.384 (0.06)

Table 6. Average running times (in seconds) for the models considered. Running times were computed for coronary artery disease (31 seeds) on the WL PPI network. Shown here are the average running times over 5 runs. Standard deviations are shown in parentheses. Note that all methods except for DIA score all nodes in the network in parallel; for DIA we computed the top 500 scores only. Experiments were carried out on a Intel(R) Xeon(R) CPU E5-2673 v3 @ 2.40GHz processor with 16 cores and 112 GiB of RAM.

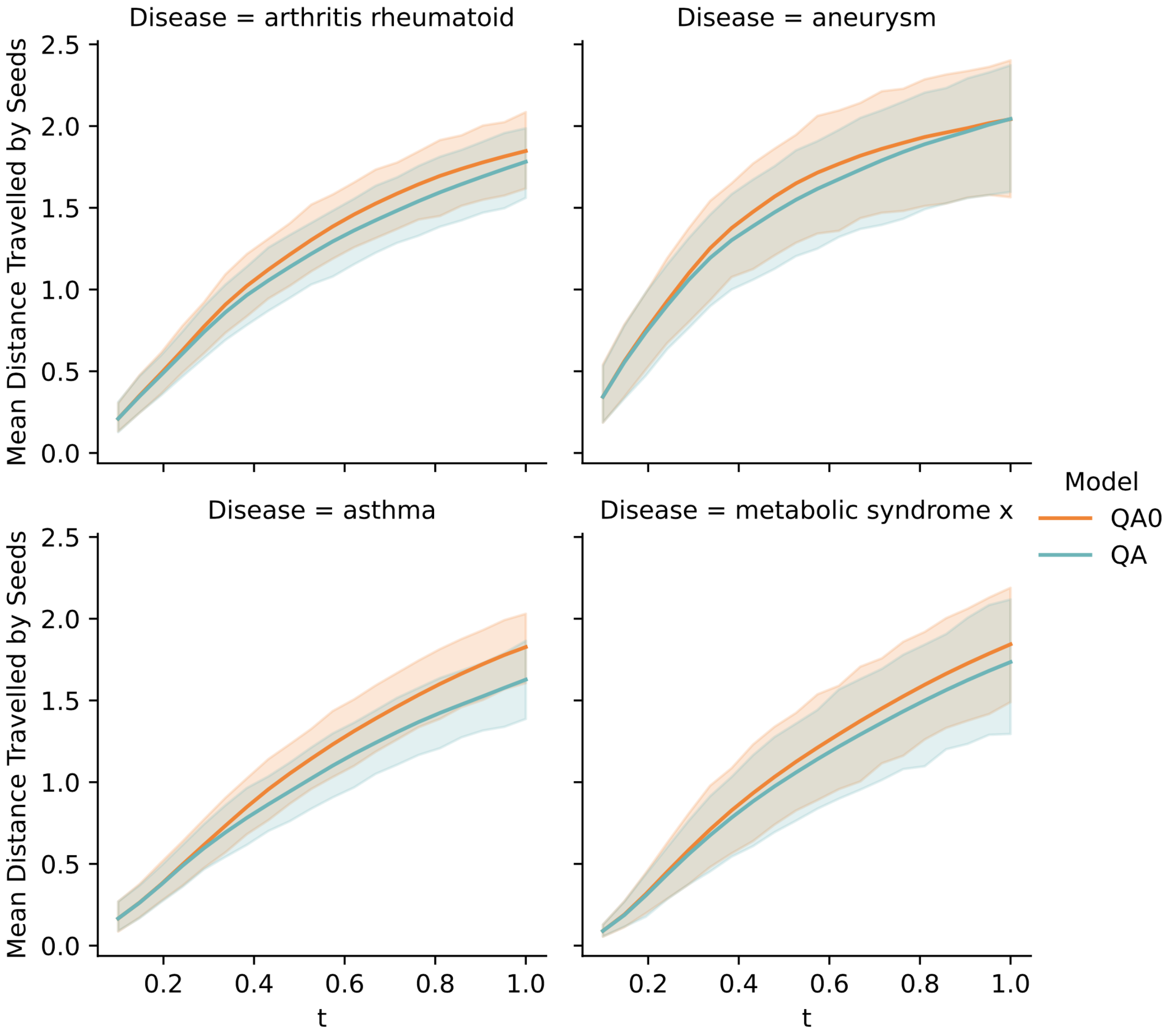

Fig. 4. Mean distance travelled for 4 diseases from the GMB disease set on the GMB PPI network.

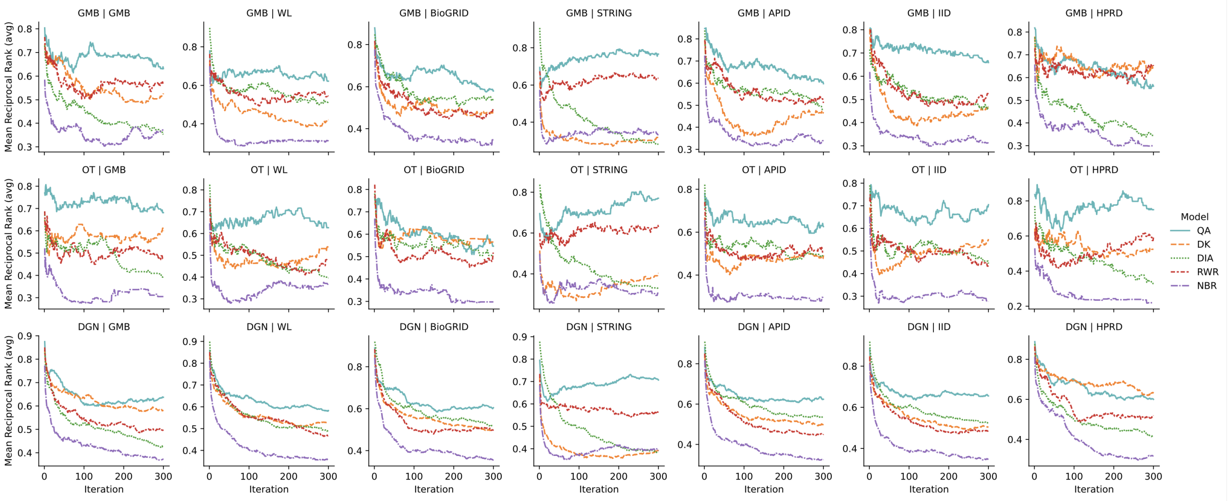

Fig. 5. Mean reciprocal ranks averaged over 10 runs and all diseases for the three disease sets (rows) and seven networks (columns).

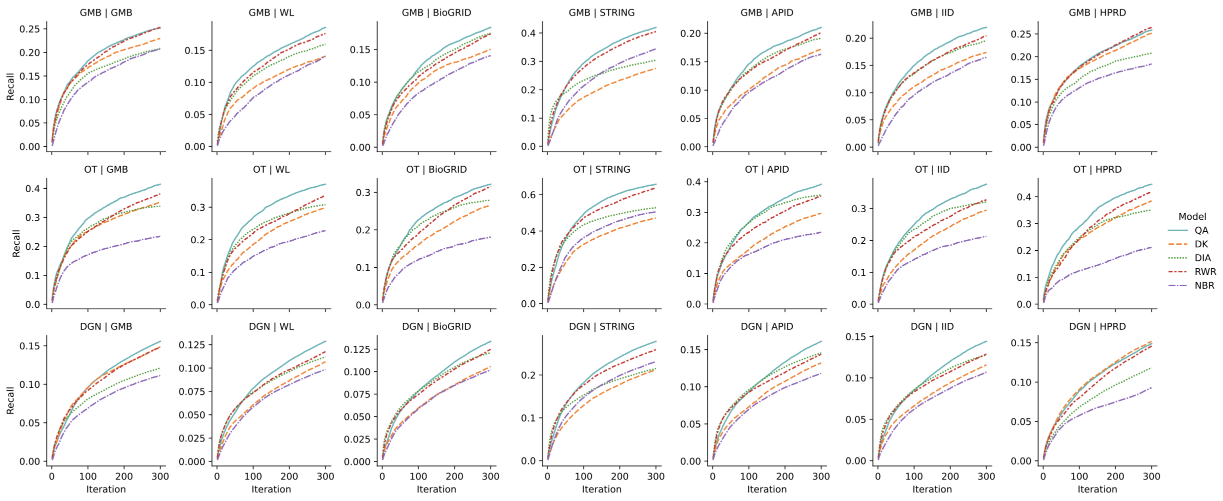

Fig. 6. Recalls averaged over 10 runs and all diseases for the three disease sets (rows) and seven networks (columns).

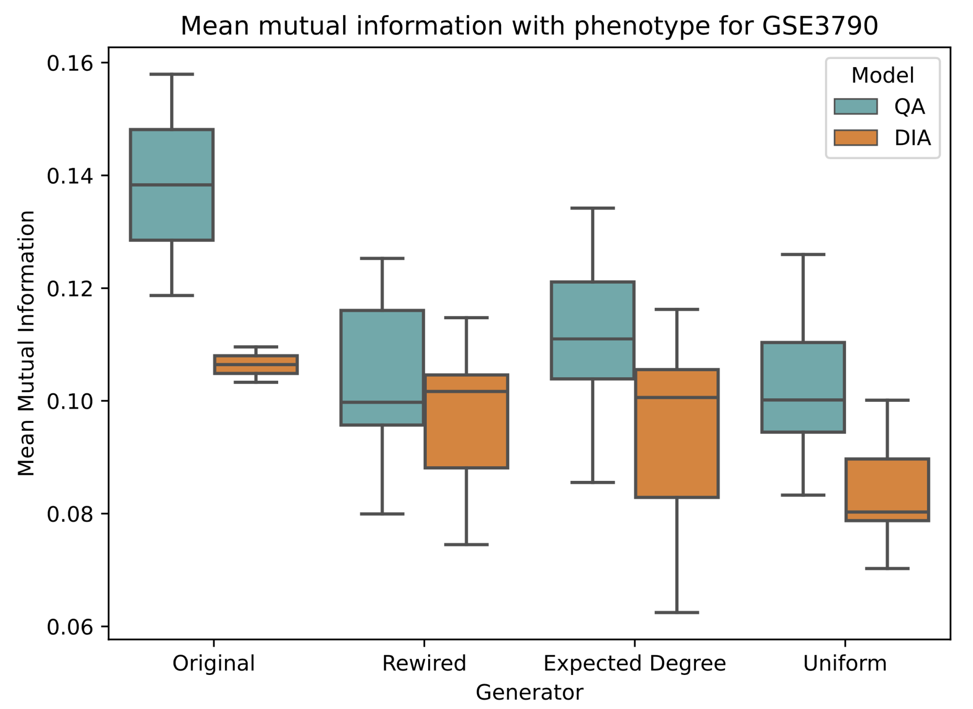

Fig. 7. Predictive performance of QA and DIA on the original and randomized networks. Mean mutual informations were averaged over the GMB and HPRD networks for the 'original' evaluations, and averaged over 10 randomized network instances for each original network. Hyperparameters for QA and DIA were chosen as in the main text.
